# Assessing the Performance and Lifetime of Cellulose Nitrate Lacquer on Silver

**DOI:** 10.3390/ma18174155

**Published:** 2025-09-04

**Authors:** David Thickett, Cathryn Harvey

**Affiliations:** English Heritage, Rangers House, London SE10 8QX, UK; cathryn.harvey@english-heritage.org.uk

**Keywords:** silver, tarnish, lacquer, Frigilene, cellulose nitrate, infra-red, FTIR, heritage science, collections care

## Abstract

Silver tarnish is a major issue in many heritage institutions. Applying lacquer is frequently used when preventive conservation approaches are limited. The service lifetime of the lacquer has a strong impact on resources and sustainability. Little systematic work has been published on this. This work explores three thresholds on lifetime—visual, reversibility, and loss of protection. It uses thermodynamic modelling to predict lacquer lifetime from aging at four temperatures. Samples on sterling silver with Frigilene lacquer were used and aging was assessed with a Bruker Alpha FTIR using external reflectance. The FTIR ratio of produced carbonyl peak to nitrate peaks was used to quantify the aging. The commonly used C-O-C peak was found to suffer from dispersion in a high proportion of samples, so could not be used in this study. The results were compared with measurements of lacquer on silver objects displayed in showcases and from store (with almost no light exposure). Spectra were obtained with the Bruker Alpha or an Inspect infra-red microscope. Autocatalytic effects through concentration of emitted nitrogen oxide gases have also been explored using diffusion tubes and gas ingress analysis. No significant concentration was observed. The thresholds were clearly established, and the model produced similar results to the natural aging studied.

## 1. Introduction

Silver tarnish removal is a resource-intensive process in many larger museums. The sulphide gases responsible are hard to remove from air or inside showcases, and lacquers are often used to slow the tarnishing process. For many silver collections, there are high demands on the aesthetics of the objects and lacquer appearance is scrutinised closely. Cellulose nitrate-based lacquers have been widely used and frequently meet curatorial approval. Of course, protective performance is essential, but cellulose nitrates are known to degrade [1,2]. Other potential drawbacks with cellulose nitrate have been highlighted [3], including potential to form silver nitrate carbonate on silver from emissions or direct contact. However, quite large numbers of analyses of silver object surfaces and silver coupons, exposed in showcase with cellulose nitrate lacquered objects, have not revealed any instances of this corrosion product. These include analyses with very sensitive methods such as electrochemical stripping and SIMS [4]. Several alternative materials have been investigated. The authors assessed twelve different materials over the past three decades. Although most performed well in terms of tarnish prevention, application, aging, reversibility and re-treatability, only two of these lacquers were considered to have suitable aesthetics by curators. Both were discontinued by their manufacturers and are no longer available. With aging, when the deterioration of properties reaches a certain unacceptable point, the lacquer needs removing and replacing. Its effective lifetime dramatically affects the resources needed to maintain a collection. The lifetime of conservation materials also has a strong impact on their sustainability and carbon footprint. If a treatment needs repeating every five years, this has much more environmental impact than every fifteen years. The lifetime of a commonly used lacquer, Frigilene FT16515 (a cellulose nitrate), has been assessed.

Three factors can potentially determine lifetime in a conservation sense:

1. The optical properties of the lacquer, as the aesthetics of many silver objects are highly valued.
2. The protective properties of the lacquer—as lacquers age, these decay and at a certain point the tarnishing rate of the silver below the lacquer will become unacceptable.
3. The reversibility of the aged lacquer. The relative greenness of solvents has been considered [5,6] and is another important consideration. Fourteen parameters to consider for conservation have been defined by the GoGreen project [7]. This process was collaboratively developed with several hundred conservation professionals. Frigilene can initially be removed with acetone [8], but as it ages, more polar solvent mixes are required. At a certain point, steam cleaning is needed to fully remove the lacquer. Full removal is essential for cleaning and relacquering. Steam cleaning has some risks to certain types of silver objects [8]. Initial work on reversibility has been reported [9].

If any of these three sets of properties drop below a certain level, the lacquer will need to be removed. But what is the level and who decides whether the level has been reached? In this work, curatorial and conservation opinions have been investigated for the optical properties. The protective properties have been assessed by exposing aged lacquer samples on silver in an accelerated experiment and determining the tarnish rate of the silver with colourimetry. Lacquer samples on silver aged at 60 °C were used in these experiments with comparison to lacquer on naturally aged objects. At present, relacquering is mainly driven by visual perception (and sometimes upcoming exhibitions). The different environmental conditions, in different rooms and even showcases, mean it is impossible to predict when a lacquer will need replacing. An accurate model would convert environmental monitoring data into predicted lacquer lifetimes and allow more efficient management of the significant resources required to maintain lacquered silver collections.

Two main mechanisms for cellulose nitrate lacquer degradation in most museums’ environments have been identified. These environments correlate with studies of moderate nitrogen content, cellulose nitrate deterioration below 100 °C, and with most UV excluded. The two mechanisms are

(1) Homolytic scission (a function of temperature and RH) and photoxidation [10,11,12]. Both of these mechanisms will lead to the loss of nitrate groups on the cellulose ring and the formation of carbonyl groups at that location. This is readily visible in the FTIR spectrum of cellulose nitrate. Frigilene is a commercial formulation with several materials in addition to cellulose nitrate present. For FTIR evaluation, the presence of phthalate plasticisers is important as they have a strong absorption at 1730 cm^−1^. However, Frigilene appears to undergo a quite rigorous quality control. FTIR spectra of fifteen batches of Frigilene purchased over 30 years has indicated very stable 1650/1730 cm^−1^ ratios with ‘as cast’ films of the new material.

(2) Aging a material above its glass transition temperature can introduce changes in behaviour, as the material is different [13]. The literature gives glass transition values between 52 and 57 °C [14]. Two measurements of Frigilene samples on steel with dynamic mechanical analysis indicated a broad transition temperature around 52 °C [15,16].

Lifetime predictions have been undertaken using aging at four temperatures; 35, 40, 45, and 50 °C. The RH was set to the average experienced by large silver collections, 45%. Environmental monitoring of eight showcases and on open display and four stores determined an average value near 45%. The FTIR ratios were assessed with AKTS sparse data software, version 7.00004, to determine the best fit rate equation, from several hundred published examples included in the software [17,18,19]. The equation was used to predict the ratios for actual showcase thermal histories. Lacquered objects in those showcases and stores were measured and the ratios compared to the predicted values.

In stores, the lacquer is subject to mainly thermal aging as they are only lit when used and blacked out otherwise. The signing out procedures for store keys, combined with spot readings, estimated light doses below 2000 lux hours per year. In showcases, the lacquer is subject to both thermal and light aging. Measured light doses (UV was below 25 µW/lumen in all showcases) were used with previous accelerated light aging [9] to estimate the FTIR ratios expected from this process. This was compared to the estimated thermal aging. The autocatalytic nature of cellulose nitrate deterioration has been postulated to be related to increasing nitrogen dioxide concentrations [1,20]. Tightly sealed showcases will exacerbate this process. Nitrogen dioxide and nitric acid concentrations in real situations were investigated. Showcases with a wide range of air exchange rates were tested, including very low values.

To assess the protective nature of aged lacquer, a form of accelerated aging of silver tarnishing is required. Accelerating the tarnish rate of silver is complicated. Relative humidity has a limited effect (about a threefold increase from 15% to 75% at 0.1 ppm hydrogen sulphide). Temperature has some effect, but retaining a hydrogen sulphide source is challenging. Increasing hydrogen sulphide or other tarnishing gas concentration can alter the kinetics in unexpected ways [21]. A method using increased air flow or polluted room air was developed [22]. This was used with thermally aged, lacquered silver pieces to assess the failure of the protective effect of the lacquer.

## 2. Materials and Methods

### 2.1. Thermal Aging and Modelling

Coupons (12 mm by 55 mm) were cut from sterling (92.5% silver, remainder copper) silver. One layer of Frigilene FR56150 lacquer was applied by brush, with 7 days drying after application.

Three coupons were placed in four Memmert ECO 260 environmental chambers (Memmert, Schwabach, Germany) at 45% RH and temperatures for 50, 45, 40, and 35 °C. The RH for aging was selected from the average value of three locations with significant amounts of lacquered silver on display. The coupons were aged in various intervals for up to 91 days.

The coupons were analysed with a Bruker Alpha FTIR spectrometer (Bruker, Coventry, UK) with external reflection. The analytical area covers most of the coupon surfaces with space at the top and bottom for handling and to enable attachment to a steel rig to present the coupons parallel to the FTIR. After FTIR analysis, coupons were replaced in the environmental chambers to continue aging.

**Figure 1:**
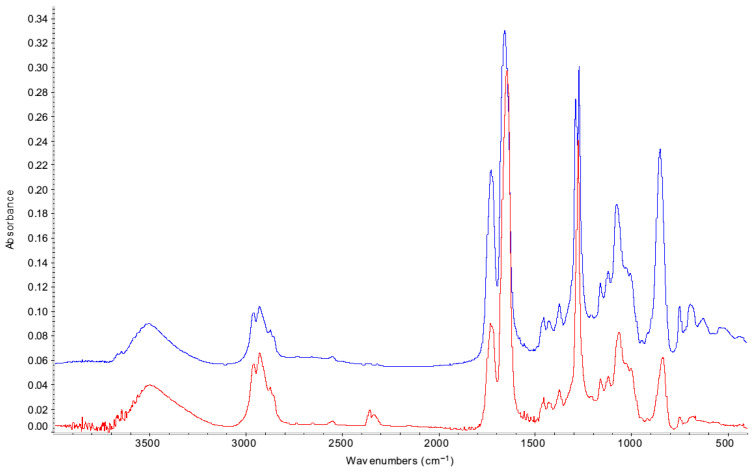
FTIR spectra of two Frigilene samples on sterling silver aged to different extents. The red spectrum is unaged Frigilene, the blue highly aged.

The spectra obtained were of good quality (Figure 1).

The main peaks are assigned in Table 1.

**Figure 2:**
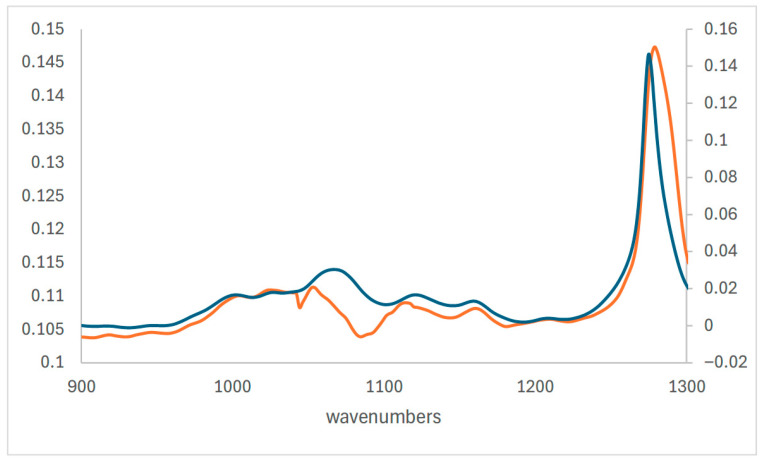
Expanded region showing dispersion effect on C-O-C peak in orange spectrum.

A significant proportion (over 15%) showed dispersion effects for the 1100 cm^−1^ peak associated with C-O-C bonds (Figure 2).

Several authors have used the ratio of the nitrate peak at around 1650 cm^−1^ or the carbonyl formed at around 1725 cm^−1^ to this peak to assess cellulose nitrate deterioration. The visible dispersion effects precluded this approach.

The ratio of peak height under the peak around 1650 cm^−1^ (nitrate) to that around 1725 cm^−1^ (carbonyl) was measured. The baseline was set at the lowest point at each end of the peaks due to some overlap. Whilst some researchers use peak area instead of peak height, the significant and changing overlap of the bands as cellulose nitrate ages would have introduced errors in the area measurement. The peak height is less affected. Other authors have used peak height ratios for cellulose nitrates and cellulose acetates [10,11,20,23]. Over half the examples in Derrick et al.’s review of FTIR in conservation used peak height for quantification [24].

This aging FTIR peak ratio data was analysed with AKTS sparse data kinetics software, v 7.00004. A subset of time intervals showing clear increases in the FTIR ration were selected for the kinetic analysis. All solid-state models were assessed and those with lowest Akaite and Bayesian weights were selected to model. This approach gives a relative weight. The weight with the most predictive value depends on the ration of the number of data points to the number of parameters [17]. In this instance, as the number of data points (57) was much greater than the number of parameters (6), the Akaite weight was given stronger value.

Coupons aged at 60 °C were used in the later parts of this work. Using results from the coupons aged at 60 °C as thresholds for the modelling performed at 35–50 °C could be problematic. Isothermal predictions at 60 °C were calculated from the modelling and compared to measured values over those time intervals. The time required to reach sufficient deterioration to cross the curatorial threshold or impact protective properties at the relatively low temperatures, determined by the glass transition temperatures, was excessive and environmental chamber access could be obtained.

### 2.2. Room Temperature Aging

Several Frigilene lacquered silver objects displayed in a series of showcases and stores were also analysed with either the Bruker Alpha spectrometer (Bruker Camdbridge, UK) or Nicolet Inspect IT microscope running of Avatar 360 bench (both now Thermo Fisher, Walham, MA, USA), with (8 analyses at different locations. Objects with large flat areas were selected to give good spectra. Objects were analysed within a year of lacquering and after several years. The showcases and stores had continuous temperature and RH monitoring with NAMAS traceable calibrated temperature and RH probes (Rotronic hygroclip probe running onto a Meaco radiotelemetry system (Meaco Measurement and Control, Newcastle-under-Lyme, UK) or Vaisala humicap running on Hanwell radiotelemetry system, Vaisala, Vantaa, Finland). Light exposure was monitoring continuously (Meaco) or using blue wool dosimeters with Minolta CR500 or 2600 D, Konica Minolta Sensing, Osaka, Japan colourimeters [25]. The measurement position was controlled with a Melinex^®^ mask (Mylar Specialty Films, Chester, PA, USA) to reduce placement errors [26] and improve the errors on the estimated light doses. A series of objects cleaned and lacquered for an exhibition, but not displayed and then returned to store, were also analysed. The lacquer had minimal light exposure.

The temperature profile from the showcase was inputted to the developed model and the ratio value after 10 years estimated. The modelled and measured values were then compared.

A comparison of the fixed focus microscope FTIR (Nicolet, Thermo Fisher Scientific, Waltham, MA, USA) results with those from the external reflection (Bruker) was undertaken. Twenty coupons from 60 °C aging were analysed with both systems. A single analysis with the external reflection and 8 spots with the microscope was conducted. Ratios were calculated and compared.

### 2.3. Visual Perception

Coupons from previous thermal aging at 60 °C [9] were examined by four curators to assess their opinion of the lacquer surface. The aging intervals were 7, 14, 21, 28, 35, and 49 days. The surfaces were inspected with microscopy to determine if white spots had appeared. FTIR spectra were run and peak ratios measured. The surface appearance was assessed by eight professionals: four curators, whose collections contained large numbers of silver objects or silver objects of high significance, and the four conservators that cared for those collections. Each was shown a total of 30 coupons under good daylight conditions and asked to say whether the surface was acceptable or if removal and relacquering would be required.

### 2.4. Protective Effect

The longer 60 °C aged coupons were exposed to the polluted atmosphere in Apsley House, central London. The coupons were placed in the inlets of the showcase air purifier system. This drew room air across the coupon surfaces. Any tarnishing was assessed with colourimetry (Minolta 2600 D, Minolta, Osaka, Japan annually calibrated by manufacturer); Raman confocal spectroscopy (Renishaw Versa, Renishaw, Wotton-under-edge, UK with 532 nm laser and ×100 lens); and some coupons with electrochemical stripping. The protective failure was assessed as when the surface yellowed (delB* > 0.4) under 6 months exposure in the flowing air. After the measurements, the coupons that were assessed as failing had their lacquer stripped with Frigilene reducer, a mix of aromatic solvents. The underlying metal colour was measured. This was to ensure the yellowing was due to tarnish and not the lacquer yellowing. The coupons were then electrochemically stripped. A 1M sodium nitrate electrolyte was used with platinum counter electrode and siler/silver chloride standard electrode and Palmsens potentiostat. Silver sulphide was estimated from the area under the peak starting at −1.2 mV versus SHE [27].

### 2.5. Nitrogen Oxides Concentration

Several showcases with extensive collections of lacquered silver were investigated. The internal concentration of nitrogen species was measured with Palmes diffusion tubes with 1 M potassium hydroxide and 10% *v*/*v* glycerol sorbents [28,29]. Both nitrogen dioxide and nitric acid have been found to present as nitrate in the ion chromatography. Measurements were undertaken when temperatures were higher, as the emission is increased under such conditions. Concentrations were also measured in the room. The IMPACT model was used to estimate the concentrations ingressing into the showcases. Air exchange rates were measured with carbon dioxide tracer gas decay, to ISO 12569 [30]. Temperatures and relative humidities measurements were as described previously. The surface areas of materials present in the showcases were assessed by simple measurement. The measured showcase nitrogen dioxide and nitric acid concentrations were compared with estimated concentrations from outside to determine any concentration occurring.

The showcase details are shown in Table 2.

## 3. Results and Discussion

### 3.1. Thermal Aging and Modelling

The FTIR peak ratios used as AKTS modelling inputs are shown in Table 3.

The best fit model was

**Figure 3:**
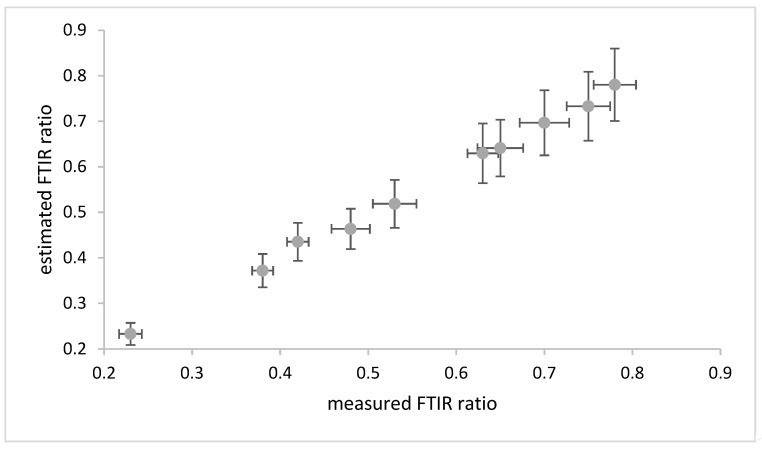
Predicted and measured FTIR ratios for aging at 60 °C.

(1) dαdt=exp79.3308−243,190.7208.314Tx 1−α52.707x a^0.802+exp−14.027−08.314Tx 1−α1 ∗ α^1.297

with values of Akaite weight of 47.3 and Bayesian weight of 45.95. The predictions for isothermal 60 °C aging are shown in Figure 3.

The predicted FTIR ratios from the 60 °C aging were very similar to the actual measured values.

### 3.2. Room Temperature Aging

**Figure 4:**
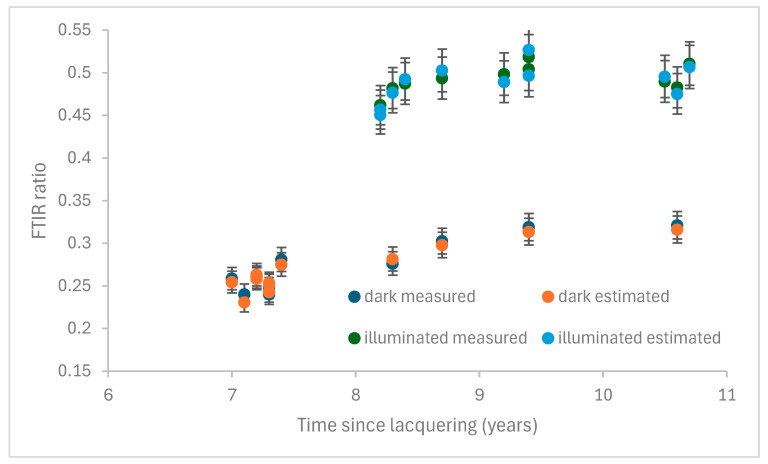
Predicted and measured FTIR ratios for natural exposure of dark and illuminated objects.

Figure 4 shows the estimated and measured peak ratios after natural exposure.

Where the time since lacquering was the same for two objects, the values closest are the estimated and measured FTIR ratios. The objects from stores had lower ratios, as expected, due to the lack of light exposure and their shorter time since lacquering. A good correlation was observed between the two sets of values in both sets of objects.

**Figure 5:**
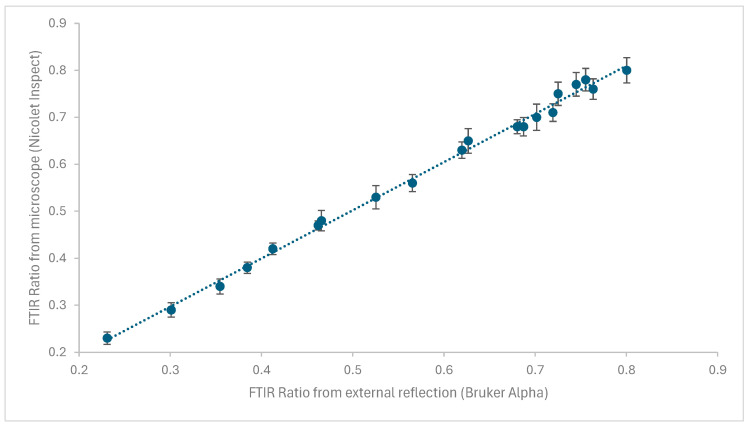
Comparison of FTIR ratios obtained with eight measurements with Nicolet Inspect IR microscope and Bruker Alpha. The dotted line is a best fit line.

Figure 5 shows the comparison of the microscope and external reflection techniques on twenty aged coupons.

A very good agreement was obtained, with each external reflection measurement lying near the mean measurement of the eight microscope measurements and within the microscope measurements ranges.

### 3.3. Visual Perception

The FTIR ratios of the coupon that was considered acceptable by curators and conservators and the one that was considered unacceptable are tabulated in Table 4.

Only certain values were produced at the time intervals used during aging at 60°. This meant some differences were 0.01 whilst others were 0.02. Whilst there is some spread of value, as expected, the professional opinions did form quite a close group of values. Overall, the conservators judged that the lacquer lifetime was reached at very slightly lower values. These values are higher (later) than those for reversibility under light aging. Once the lacquer FTIR ratio reached 0.48, the lacquer was no longer fully soluble in acetone. Stronger solvents would still remove the lacquer before steam cleaning would be required. Some objects that had lacquer FTIR ratios measured were also examined and ranked. Results agreed with the coupon assessments, although the number of objects was lower, therefore resulting in greater intervals.

### 3.4. Nitrogen Oxides Concentration

**Figure 6:**
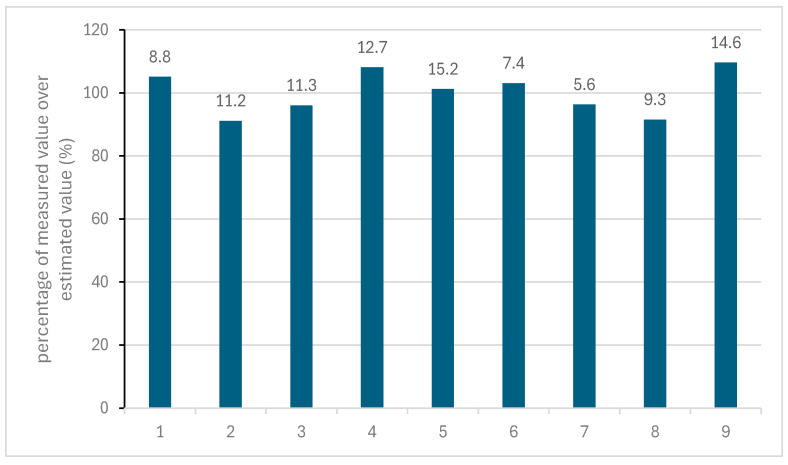
Nitrate concentrations measured in showcases.

The percentages of nitrate detected, divided by the estimated room value (from room measurement and showcase parameters), is shown in Figure 6. The figure also contains the measured showcase concentrations in ppb as labels.

The ratios were close to 100, with no more than a 10% variation. Slightly more values (5) were higher than 100%, then lower. If any elevated concentration of nitrogen dioxide or nitric acid was occurring, it was only slightly above the estimated value permeating in from the room. The highest extra concentration was 1.4 ppb on an estimated showcase concentration of 14.6 ppb. There are always uncertainties with any modelled data, but the process has been found to work reliably with previous showcase data [31].

### 3.5. Protective Effect

**Figure 7:**
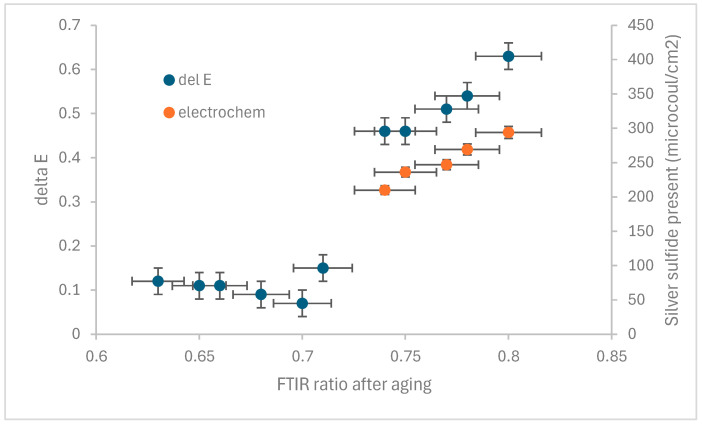
Change in b* and amount of silver sulphide present after 6 months exposure versus FTIR ratio.

The FTIR ratios of the coupons and their increase in b* calculated from colourimetry are shown in Figure 7.

There is a clear failure point in the protective effect of the lacquer when the FTIR ratio exceeds 0.71. Once 0.74 has been reached, the yellowing rate increases with increasing FTIR ratio. The colour measurements after the lacquer was stripped were within 0.05 of those taken from the lacquered surface. No silver sulphide was detected with Raman on the coupons with ratio below 0.71 after exposure. Silver sulphide was detected on those above 0.74. This was confirmed by detection of sulphide in the electrochemical stripping. The stripping errors are smaller and mainly driven in inaccuracy in measuring the stripped area, which was conducted by simple measurement with a ruler. After another five months exposure, Raman did not detect silver sulphide on any of the coupons with an FTIR ratio below 0.71.

## 4. Conclusions

Thermal aging and modelling Frigilene lacquer deterioration using the 1725 to around 1650 cm^−1^ band ratio successfully generated a model. Predictions for isothermal aging at 60 °C correlated well with experimental measurements. The model predictions correlated well with measurements from objects that had both mainly thermal exposure and combined thermal and light exposure.

Previous work indicated that light aging achieved a FTIR ratio threshold of 0.41 before stronger solvents than acetone would be needed for full removal. The lacquer was visually unacceptable at higher values between 0.65 and 0.71, depending on the curator or conservator in question. The loss of protective effect does not occur until further deterioration above 0.73.

There appears to be little evidence of concentrated nitrogen dioxide or nitric acid atmospheres in showcases with lacquered silver collections. These included some very low air exchange showcases.

The model, combined with the thresholds, allows for a prediction of the lifetime of this important lacquer from environmental measurements. This is an important ability to help manage silver collections. The model will be integrated into the online spreadsheet developed to predict the sustainability impacts of managing silver heritage collections [9].

## Figures and Tables

**Table 1:** Main peak assignments for Frigilene lacquer.

3462	O-H
2964	C-H
1728	C=O initially from phthalate, then also degradation product
1636	NO_2_
1274	NO_2_
1050	COC
829	NO

**Table 2:** Showcase details.

Arbitrary Number	Length (m)	Width (m)	Height (m)	Lacquered Silver Area	Air Exchange Rate (day^−1^)	Materials, Top, Bottom, Sides (Back)
1	1.0	1.0	2.4	0.43	0.02	Metal, fabric, glass (fabric)
2	1.0	1.0	2.4	1.54	0.03	Metal, fabric, glass (fabric)
3	1.5	1.0	1.5	0.20	0.21	Wood, fabric, glass (wood)
4	1.5	1.0	1.5	1.01	0.32	Wood, fabric, glass (wood)
5	1.5	1.0	1.5	0.50	0.40	Wood, fabric, glass (wood)
6	2.2	0.8	0.4	4.13	0.42	Glass, fabric, glass
7	1.5	0.6	0.6	2.12	0.60	Glass, fabric, glass
8	1.5	0.6	0.6	1.09	0.70	Glass, fabric, glass
9	0.8	0.8	2.0	0.10	0.80	Metal, fabric, glass

**Table 3:** Measured FTIR ratios after thermal aging at 45% RH.

Temperature (°C)	Time (days)	Ratio	Temperature (°C)	Time (days)	Ratio
50	0	0.220	40	0	0.22
50	11.691	0.277	40	11.611	0.226
50	11.691	0.282	40	11.611	0.225
50	11.691	0.282	40	11.611	0.226
50	18.732	0.304	40	30.039	0.234
50	18.732	0.297	40	30.039	0.241
50	27.448	0.346	40	30.039	0.236
50	27.448	0.336	40	47.262	0.247
50	27.448	0.346	40	47.262	0.245
50	36.312	0.386	40	47.262	0.241
50	36.312	0.396	40	70.046	0.277
50	36.312	0.404	40	70.046	0.282
50	43.896	0.433	40	70.046	0.288
50	43.896	0.423	40	91.317	0.337
50	43.896	0.431	40	91.317	0.327
50	51.174	0.495	40	91.317	0.331
50	51.174	0.493	45	0	0.22
50	54.775	0.530	45	11.850	0.241
50	54.775	0.532	45	11.850	0.239
35	0	0.22	45	11.850	0.232
35	91.051	0.223	45	23.832	0.279
35	91.051	0.219	45	23.832	0.278
35	91.051	0.221	45	23.832	0.286
			45	36.411	0.309
			45	36.411	0.303
			45	36.411	0.305
			45	53.016	0.375
			45	53.016	0.379
			45	53.016	0.370
			45	62.853	0.425
			45	62.853	0.418
			45	72.532	0.517
			45	72.532	0.511
			45	72.532	0.503

**Table 4:** Curator and conservator assessment of the point at which lacquer becomes ascetically unacceptable.

Site	Person	Acceptable FTIR Ratio	Unacceptable FTIR Ratio
Apsley House	Curator	0.63	0.65
	Conservator	0.68	0.70
Audley End House	Curator	0.68	0.70
	Conservator	0.65	0.66
Brodsworth Hall	Curator	0.70	0.71
	Conservator	0.68	0.70
Osbourne House	Curator	0.66	0.68
	Conservator	0.65	0.66

## Data Availability

The original contributions presented in this study are included in the article. Further inquiries can be directed to the corresponding author.

## Abbreviations

The following abbreviations are used in this manuscript:

FTIR	Fourier Transform Infra-Red
